# Supplementary material for: Prefrontal parvalbumin interneurons require juvenile social experience to establish adult social behavior
Source: Nat Commun. 2020 Feb 21;11:1003. doi: 10.1038/s41467-020-14740-z (PMC7035248; doi:10.1038/s41467-020-14740-z)
Supplement: Supplementary file 1 — Supplementary Information [file 41467_2020_14740_MOESM1_ESM.pdf]

Supplementary Information

Bicks et al

Prefrontal parvalbumin Interneurons require juvenile social experience to establish adult social behavior

Supplementary Figures

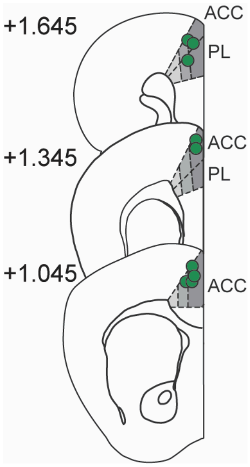

Supplementary Figure 1 (related to Fig. 1): Ferrule placement and GCaMP6f injection sites in group housed PV-Cre mice for fiber photometry. Ferrule placement and proximity to injection sites were confirmed from all mice n =9 mice. Grey area shows dorsal medial PFC injection target area and layers.

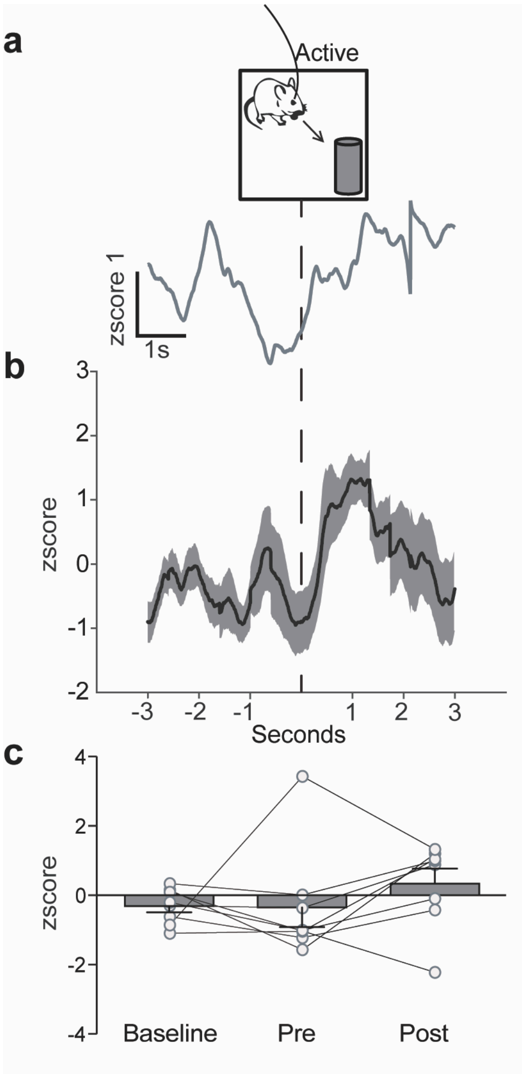

Supplementary Figure 2 (related to Fig. 1): Active object exploration in group housed PV-Cre mice during fiber photometry imaging. a) Top: Active exploration, defined as approach to the object was manually scored. Bottom: Example trace showing no change in GCampf6f signal in dmPFC-PVIs preceding or during active object exploration. **b)** Mean and +/- SEM graph smoothed signals including all animals. **c)** There was no difference comparing baseline (-3 to -2 sec before active initiation) pre (-1 to 0 sec) and post (0-1 sec) across all animals (One-way repeated measures ANOVA, $F(2,7) = 1.23$, $p = 0.43$, $n = 8$ mice). All error bars reflect +/- SEM.

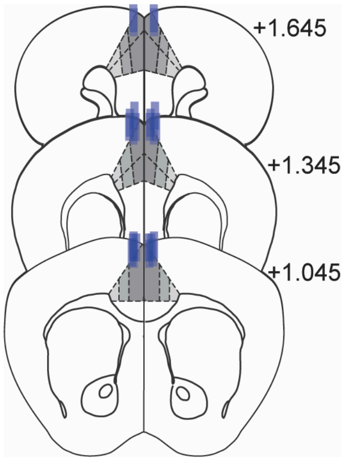

Supplementary Figure 3 (related to Fig. 2): Cannula, location for wireless optogenetic experiments. LED location and proximity to viral injection was validated in all mice, n = 12 mice. Grey area shows dorsal medial PFC injection target area and layers.

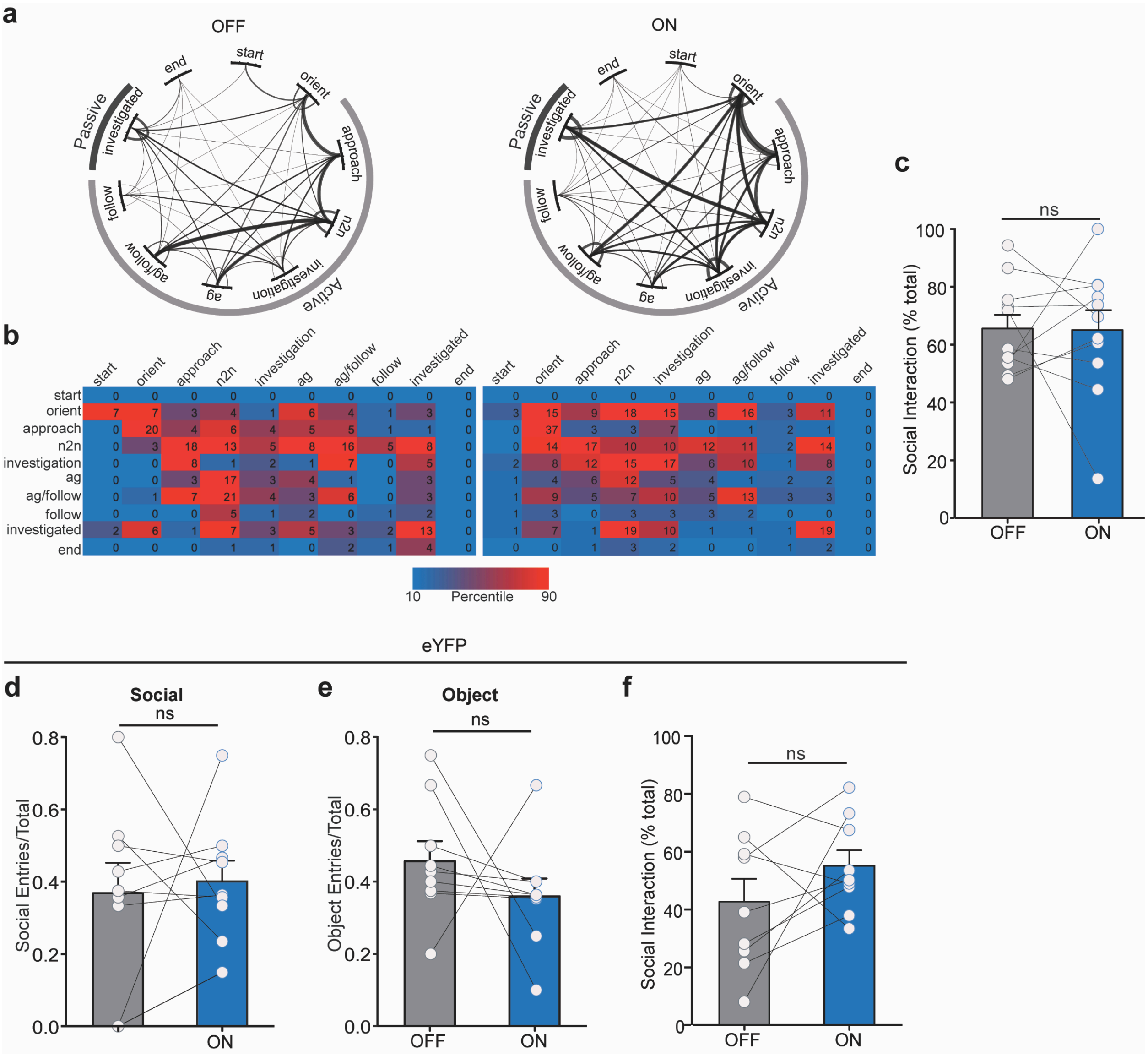

Supplementary Figure 4 (related to Fig.2) : Behavioral sequence or social interaction percent total between on and off conditions a) Behavior transitions between behavior categories were compared between light 'OFF' and light 'ON' conditions. No significant differences were found between behavior joint matrices of the two groups ($p < 0.05$). **b)** Transition matrices showing frequency of transitions between behaviors. Direction of transitions is visualized from column to row. Color scale shows percentile. **c)** Percent social interaction was not different between on and off conditions (paired t-test, $t = 0.07$, $df = 10$, $p = 0.95$, $n = 11$ mice). **d-f)** Mice injected with eYFP did not show an effect of light activation in the 3-chamber test in **d)** social entries (Social entries: paired t-test, $t = 0.29$, $df = 8$, $p = 0.78$, $n = 9$ mice), **e)** object entries (Object entries: paired t-test, $t = 0.98$, $df = 8$, $p = 0.36$, $n = 9$ mice), or **f)** in percent social interaction throughout the testing phase. (Social interaction percent total, $t = 1.33$, $df = 8$, $p = 0.22$, $n = 9$ mice). All error bars reflect +/- SEM.

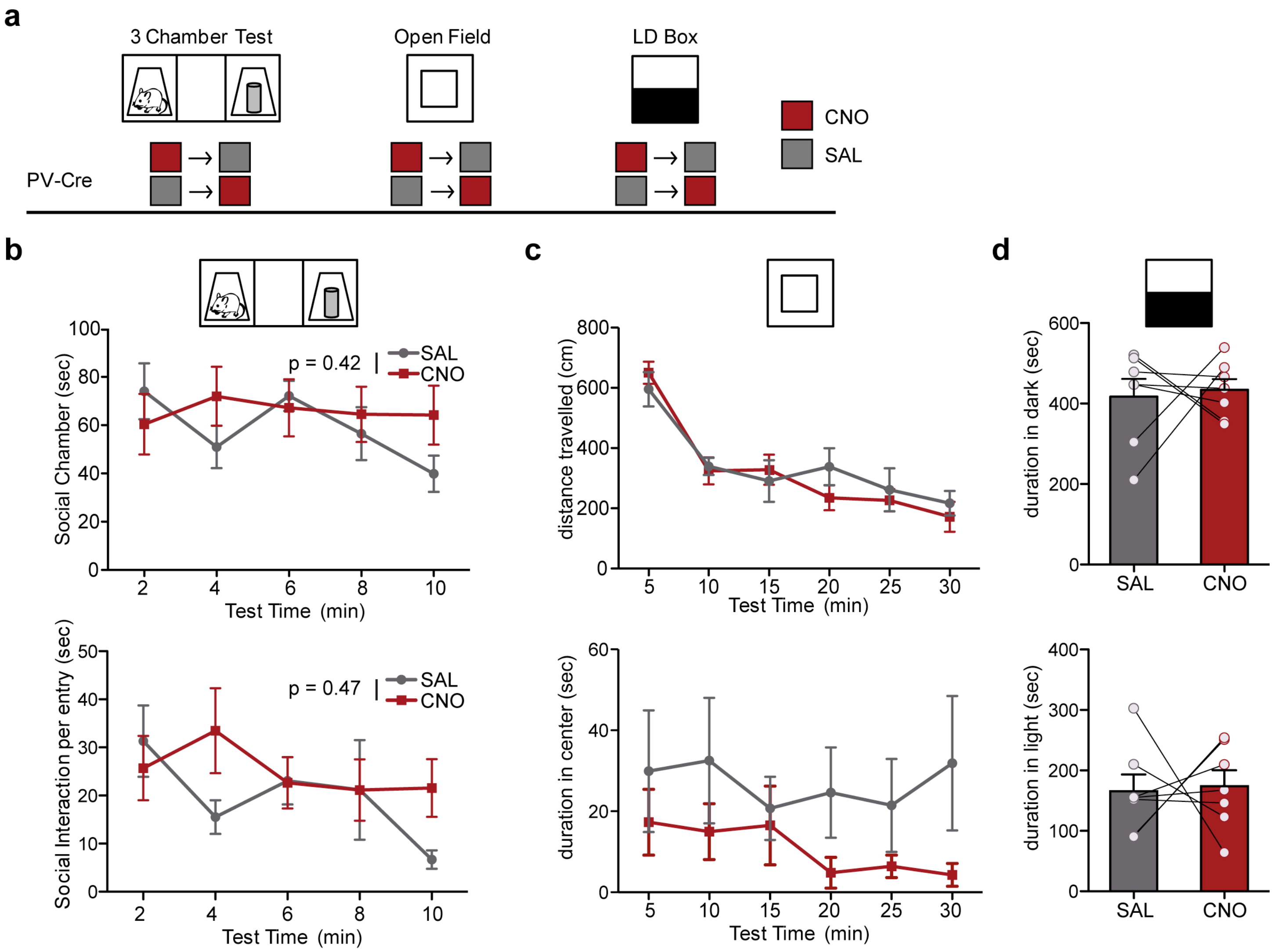

Supplementary Figure 5 (related to Fig. 3): CNO administration in the absence of viral iDREADD expression does not affect social behavior. a) Adult PV-Cre mice wre intraperitoneally injected with CNO (10 mg/kg) or SAL in a counter-balanced fashion 30 min prior to being tested in a 3-chamber test for sociability, and open field test, or in a Light-Dark (LD) box test. **c)** Top: Time spent in the social chamber was not significantly different when mice were treated with CNO compared with SAL (2-way repeated measures ANOVA, effect of drug, $F(1,8) = 0.57$, $p = 0.47$, $n = 9$ mice). Bottom: Time spent in social interaction per chamber entry was not significantly different when mice were treated with CNO compared with SAL (2-way repeated measures ANOVA, effect of drug, $F(1,8) = 0.73$, $p = 0.42$, $n = 9$ mice). **d)** Top: Distance travelled in an Open Field arena was not significantly affected by drug treatment (2-way repeated measures ANOVA, effect of drug, $F(1,7) = 0.12$, $p = 0.74$, $n = 8$ mice). Bottom: Time in center in an Open Field arena was not significantly affected by drug treatment (2-way repeated measures ANOVA, effect of drug, $F(1,7) = 1.63$, $p = 0.24$, $n = 8$ mice) **e)** Top: Time spent in light in a Light-Dark Box was not affected by drug treatment (Paired t-

test, t = 0.25, df = 6, p = 0.81, n = 7 mice). Bottom: Time spend in dark was not affected by drug treatment (Paired t-test, t = 0.15, df = 6, p = 0.88, n = 7 mice). All error bars reflect +/- SEM.

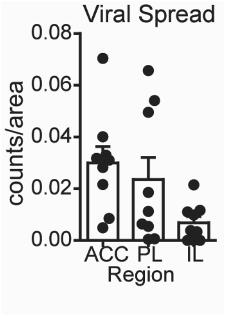

Supplementary Figure 6 (related to Fig. 3): Viral quantification in iDREADD animals. Viral spread across the dmPFC showing expression primarily in dorsal PFC (ACC and PL) and some restricted expression in IL (n = 9 mice). All error bars reflect +/- SEM.

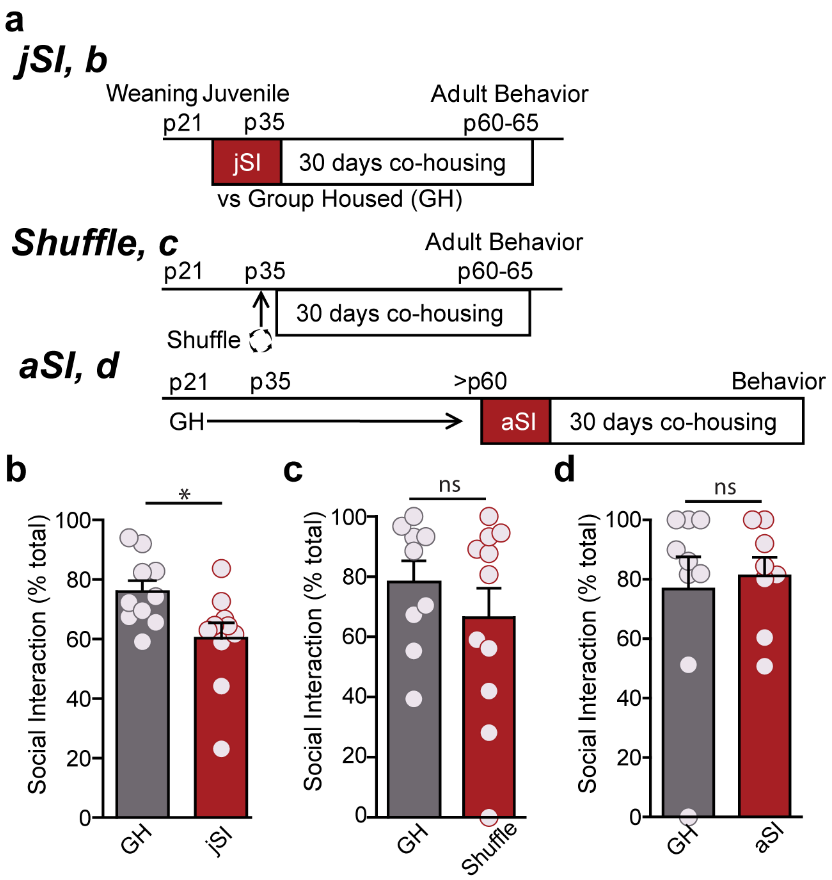

Supplementary Figure 7 (related to Fig. 4): Juvenile social isolation but not 2 week adult social isolation induces long-lasting adult social deficits. a) Schema of behavioral manipulations. **b)** Mice were weaned at p21 into isolation for 2 weeks and were re-housed with age- sex- and strain- matched males at p35 (juvenile social isolation, jSI) or were group housed from weaning (GH). In adulthood (p60-65) mice were tested in a 3-chamber test for sociability for 2 minutes. jSI mice show persistent decreases in sociability compared with controls (percent time in social investigation), student's t-test, $t = 2.47$, $df = 18$, $p = 0.02$. $n = 10$ mice. **c)** This deficit was not seen in mice that were placed with unfamiliar age-, sex-, and strain-matched cagemates (shuffled) at p35 (percent time in social investigation), student's t-test, $t = 0.94$, $df = 18$, $p = 0.36$, $n = 9$ mice GH, $n = 11$ mice Shuffle. **d)** Adult isolated mice (aSI) were isolated for 2 weeks in adulthood (>p60) and then were re-housed with age- sex- and strain- matched males for 30 days, followed by testing in a 3-chamber test for sociability. aSI mice did not exhibit any social deficits compared with GH controls, student's t-test, $t = 0.34$, $df = 15$, $p = 0.74$, $n = 10$ mice GH, $n = 9$ mice aSI. All error bars reflect +/- SEM.

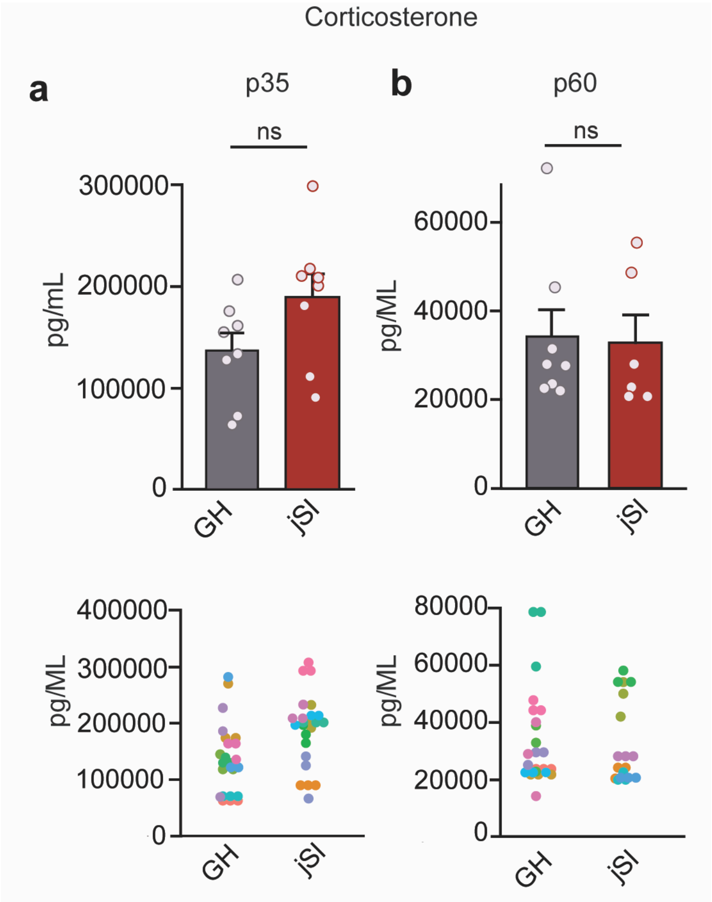

Supplementary Figure 8 (related to Fig. 4): Juvenile social isolation does not significantly alter corticosterone levels. Mice were weaned at p21 and either isolated for two weeks (jSI) or weaned into group housing (GH) and then were either (left) euthanized at **a)** p35 to collect truck blood, or **b)** re-housed at p35 and then euthanized at p60 (right). There was a non-significant trending increase in trunk blood corticosterone levels tested using an ELISA (Invitrogen) at **a)** p35 (Linear Mixed Model, effect of treatment $t = 1.96$, $df = 16$, $p = 0.07$, 8 GH mice, 8 jSI mice, triplicate values for each animal shown in bottom panel), however this increase was not maintained in p60 animals (Linear Mixed Model, effect of treatment $t = -0.167$, $df = 14$, $p = 0.87$, 8 GH mice, 6 jSI mice, triplicate values for each animal shown in bottom panel). All error bars reflect +/- SEM.

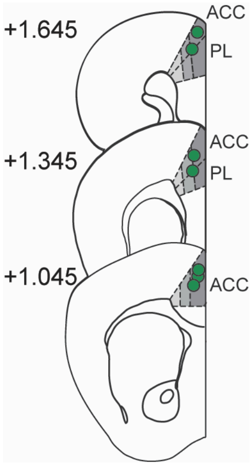

Supplementary Figure 9 (related to Fig. 4): **Ferrule placement and GCaMP6f injection sites in juvenile socially isolated mice PV-Cre mice for fiber photometry.** Ferrule placement and proximity to injection sites were confirmed from all mice, n =8 mice.

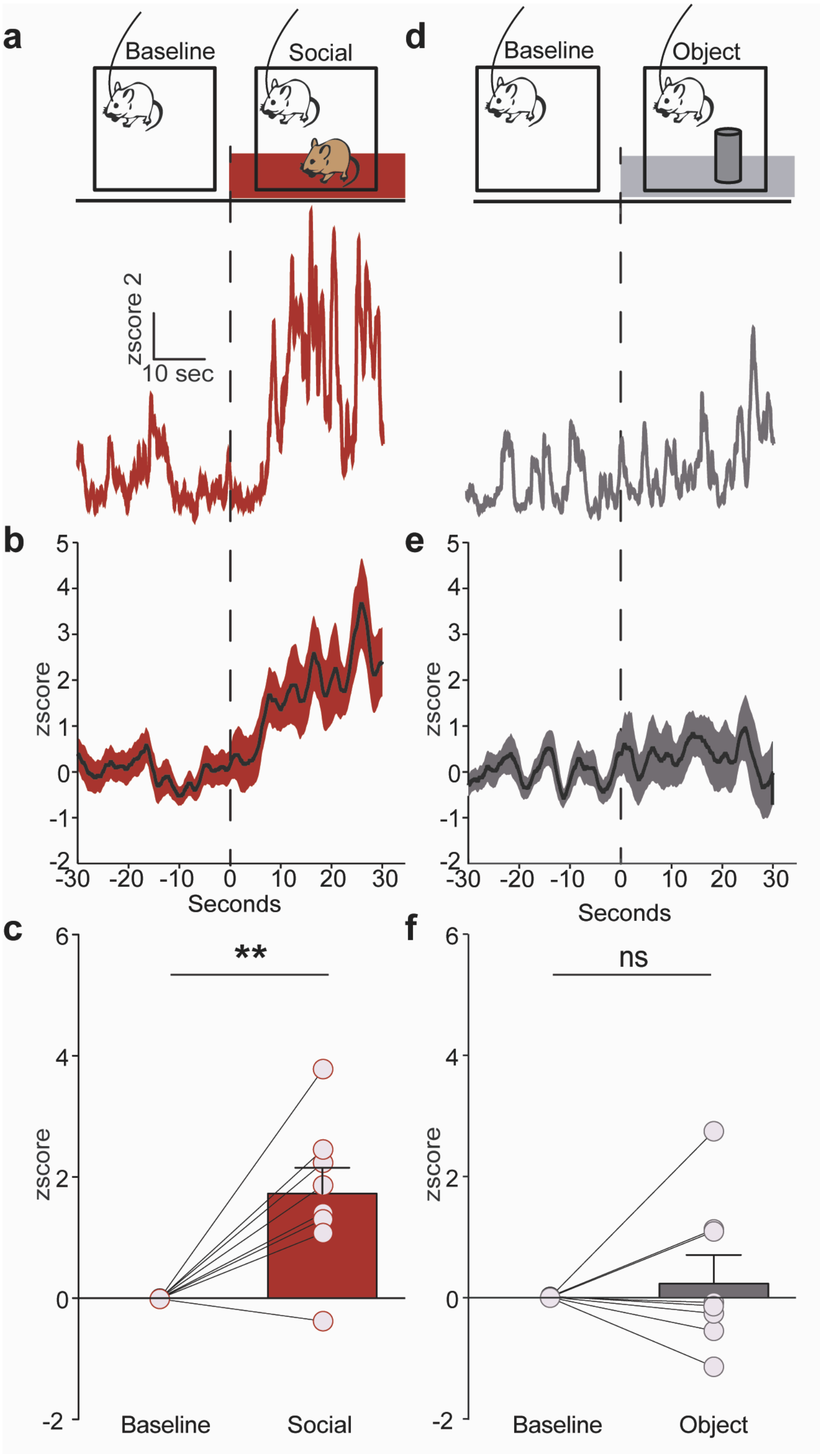

Supplementary Figure 10 (related to Fig. 4): Social exposure, but not object exposure, increases GCaMP6f in dmPFC-PVIs of jSI mice. a) GCaMP6f signals of dmPFC-PVIs in jSI mice show increased mean z-score 30 sec after introduction of a novel mouse (**a-c**), but not a novel object (**d-f**). **a,d)** Example GCaMP signal traces from social or object exposed **b,e)** Mean and +/- SEM smoothed signal **c,f)** Mean dmPFC-PVI GCaMP6f signal for each mouse comparing baseline and post stimulus introduction for social (**c**) and object

(**d)** (Wilcoxon signed rank test, social: *p = 0.02, n = 8 mice, object: p = 0.94, n = 8 mice). All error bars reflect +/- SEM.

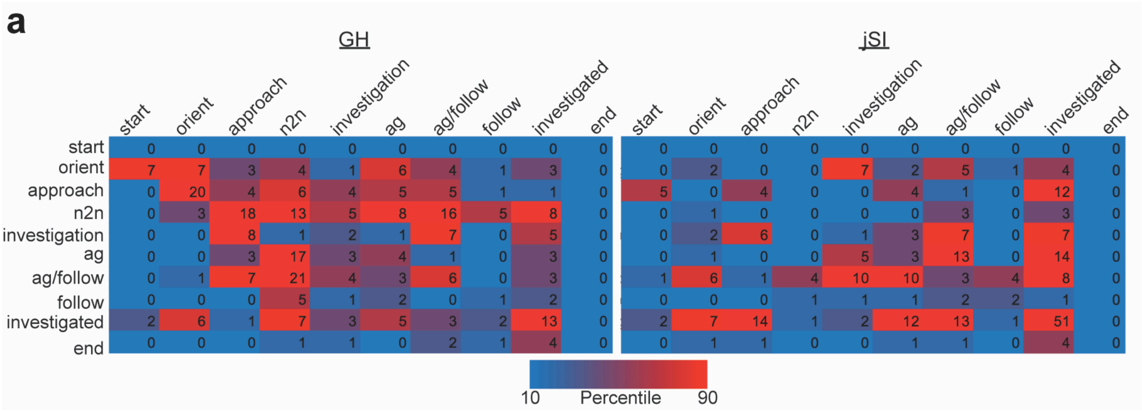

Supplementary Figure 11 (related to Fig. 4): Behavioral transition Matrices for jSI and GH mice. a) Transition matrices showing frequency of all transitions between behaviors during reciprocal interaction. Direction of transitions is visualized from column to row. Color scale shows percentile.

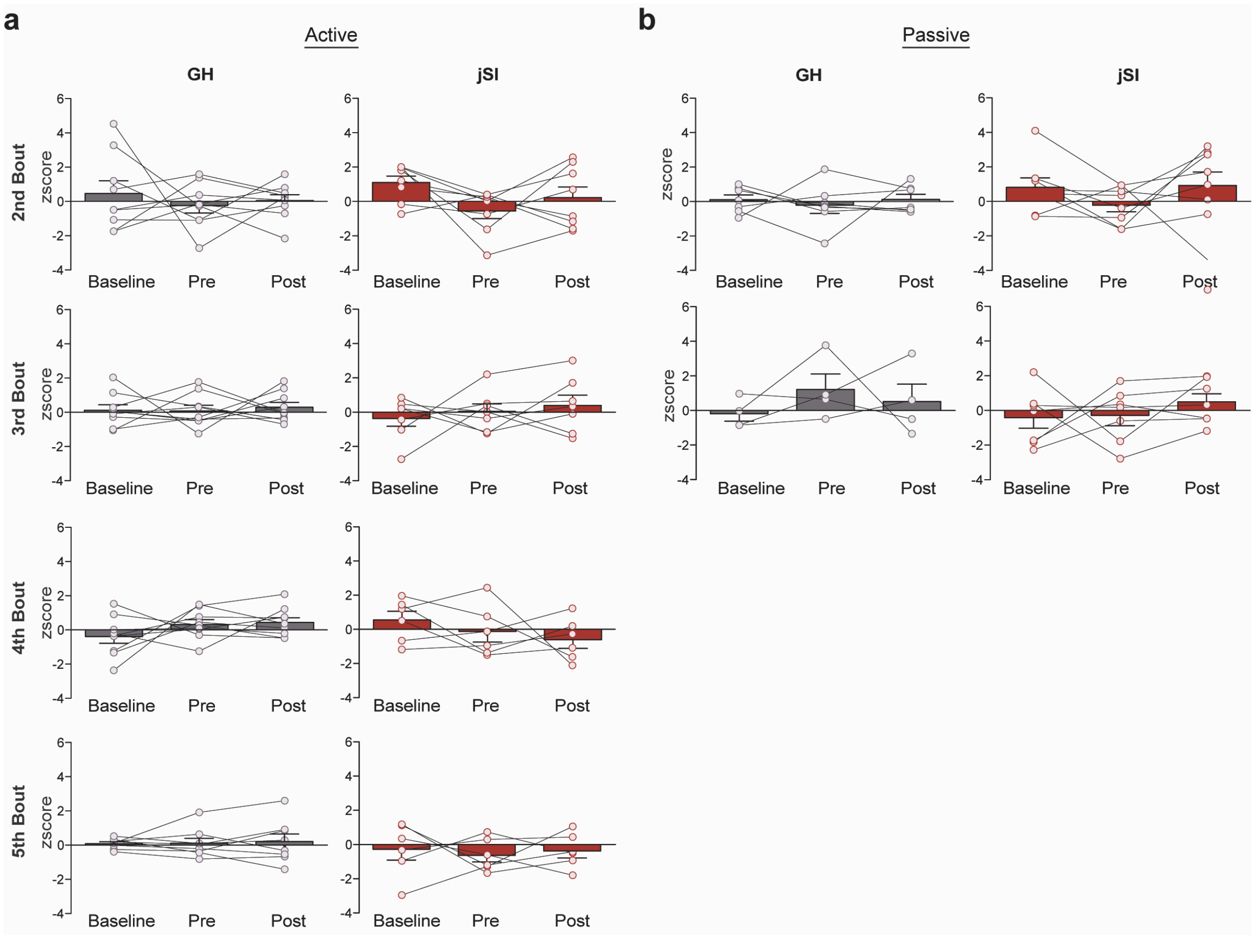

Supplementary Figure 12 (related to Fig. 4): All active and passive bouts of PV-Cre mice during fiber photometry imaging. a) Active bouts 2-5 do not show significant changes between baseline, pre, or post behavior initiation in GH or jSI mice. (One-way Repeated Measures ANOVA, 2nd Active GH: $F(2,8) = 0.32$ $p = 0.73$, 2nd Active jSI: $F(2,7) = 3.08$, $p = 0.08$; 3rd Active GH: $F(2,8) = 0.01$, $p = 0.90$, 3rd Active jSI: $F(2,6) = 0.58$, $p = 0.58$, 4th Active GH: $F(2,8) = 1.43$, $p = 0.27$, 4th Active jSI: $F(2,5) = 0.90$, $p = 0.44$, 5th Active GH: $F(2,7) = 0.07$, $p = 0.93$, 5th Active jSI: $F(2,5) = 0.11$, $p = 0.90$). **b)** Passive bouts 2-3 do not show significant changes between baseline, pre, or post behavior initiation in GH or jSI mice. (One-way Repeated Measures ANOVA, 2nd Passive GH: $F(2,6) = 0.20$, $p = 0.82$, 2nd Passive jSI: $F(2,7) = 0.83$, $p = 0.46$, 3rd Passive GH: $F(2,3) = 0.56$, $p = 0.60$, 3rd Passive jSI: $F(2,6) = 0.73$, $p = 0.50$). All error bars reflect +/- SEM.

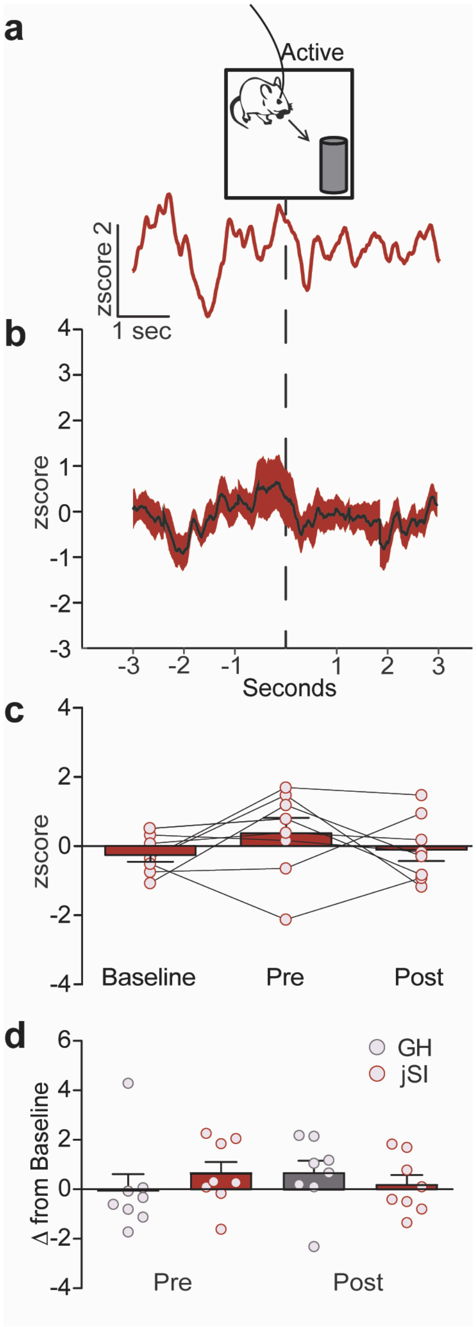

Supplementary Figure 13 (related to Fig. 4): **Active object exploration in jSI PV-Cre mice during fiber photometry imaging. a)** Top: Active exploration, defined as approach to the object was manually scored. Bottom: Example trace showing no change in GCampf6f signal in dmPFC-PVIs preceding or during active object exploration. **b)** Mean and +/- SEM graph smoothed signals including all animals. **c)** There was no difference comparing baseline (-3 to -2 sec before active initiation) pre (-1 to 0 sec) and post (0 to 1 sec) across all animals (One-way repeated measures ANOVA, $F(2,7) = 1.00$, $p = 0.39$, $n = 8$ mice). **d)** Change in z-score from baseline in the first active object bout shows no change in either group housed (GH) or jSI mice in

either the pre or post window (Wilcoxon Signed Rank Test, GH Pre: $p = 0.25$, jSI Pre: $p = 0.15$, GH Post: $p = 0.20$, jSI Post: $p = 0.84$). All error bars reflect +/- SEM.

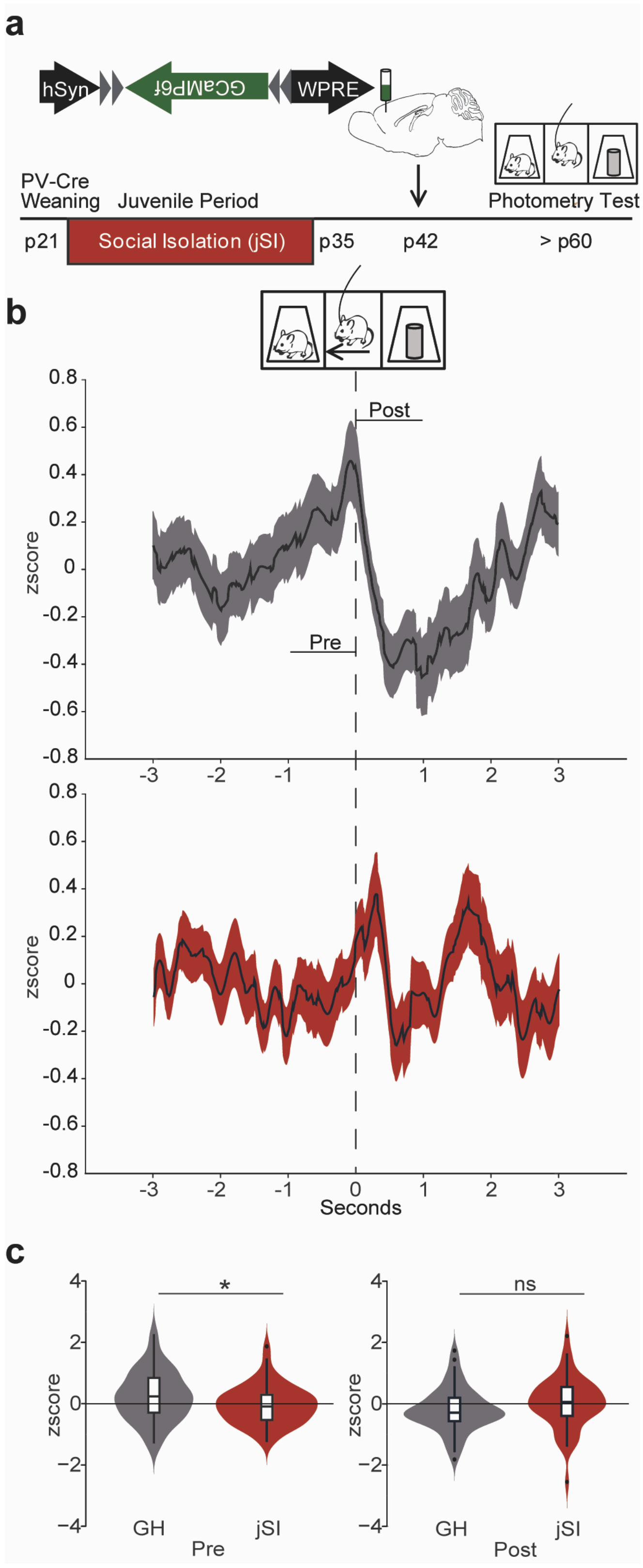

Supplementary Figure 14 (related to Fig. 4): GH, but not jSI mice show increased activity prior to social chamber entries in a 3-chamber test. a) Timeline showing juvenile social isolation (jSI) vs. group housing (GH), ddmPFC viral injection at p42, and photometry testing in the 3-chamber test in adulthood. **b)** Average

trace of all social chamber entries in GH (tope) and jSI (bottom) mice with +/- SEM. **c)** The ‘pre’ window (left panel), one second prior to a social chamber entry shows a significantly higher mean z-score in GH compared with jSI mice (Pre: Student’s t-test, $t = 2.00$, $df = 1.06$, $p < 0.05$). The ‘post’ window (right panel), one second following a social chamber entry shows no significant differences in mean z-score between GH and jSI mice (Post: Student’s t-test, $t=1.54$, $df = 106$, $p = 0.13$). n = 6 mice per group, chamber entries were bootstrapped to match across conditions, n = 54 entries per group. Boxplots within violin plots represent the interquartile range.

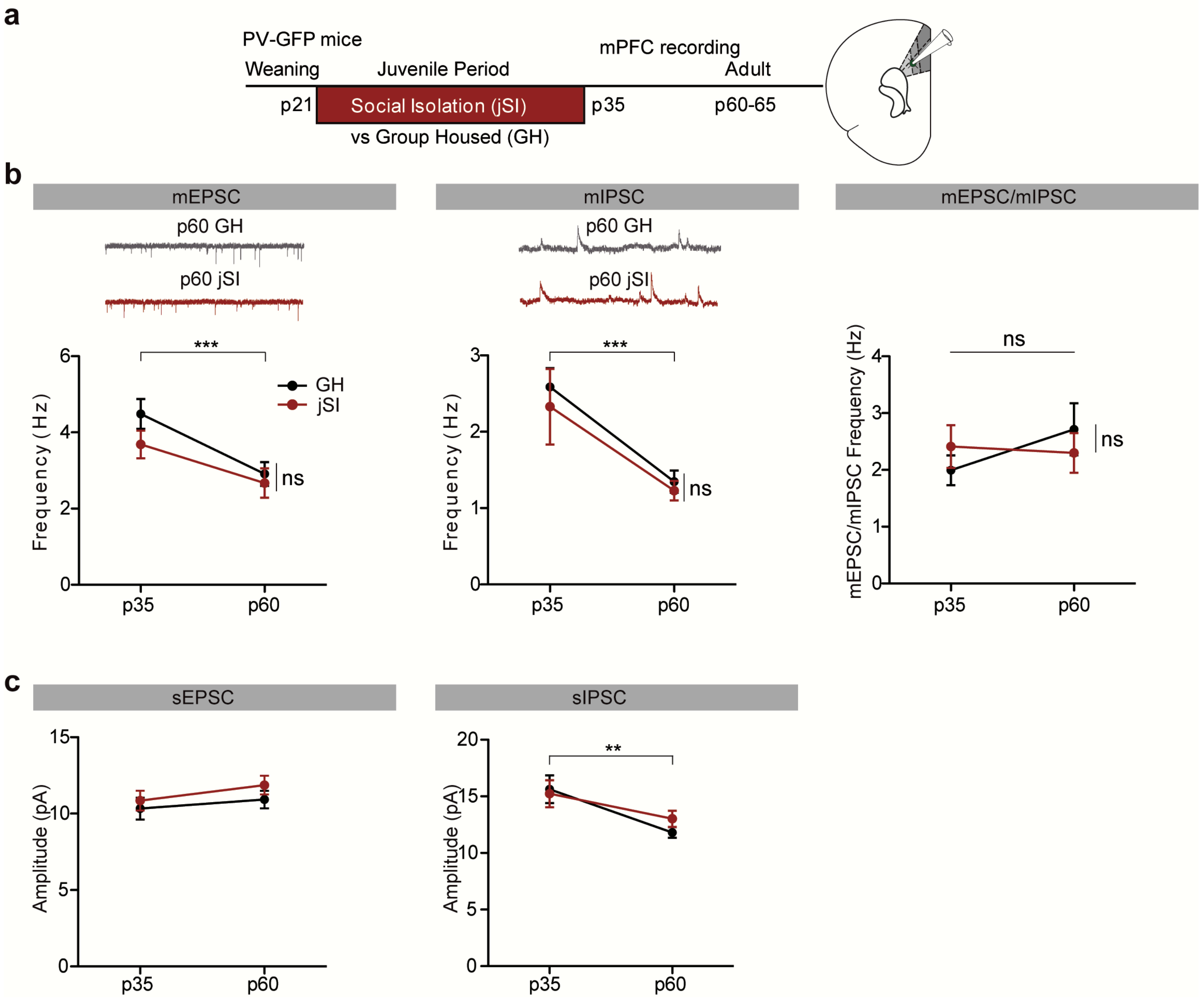

Supplementary Figure 15 (related to Fig. 5): Normal miniature and spontaneous amplitude of EPSC and IPSC of dmPFC-PVIs following jSI. a) Timeline showing juvenile isolation (jSI) vs. group housing (GH) and adult dmPFC recording in PV-GFP mice. **b)** There were no significant differences in mEPSC, mIPSC, or the ratio of mEPSC/mIPSC frequency between jSI and GH mice, however, there was a significant effect of age, indicating a decrease in frequency across development from p35 to p60. There was no effect of age on the ratio of mEPSC/mIPSC frequency. (mEPSC frequency: 2-way ANOVA, effect of housing, $F(1,70) = 2.03$, $p = 0.16$, effect of age, $F(1,70) = 12.57$, ***$p < 0.001$, mIPSC frequency: 2-way ANOVA, effect of housing, $F(1,70) = 0.43$, $p = 0.51$, effect of age, $F(1,70) = 16.67$, ***$p < 0.001$. mEPSC/mIPSC frequency ratio: 2-way ANOVA, effect of housing, $F(1,70) = 0.00$, $p = 0.99$, effect of age, $F(1,70) = 0.69$, df = 1, $p = 0.41$, n=18 cells from 5 mice (adult GH), n=20 cells from 6 mice (adult jSI) n = 18 cells from 5 mice (p35 GH), n = 18 cells from 5 mice

(p35 jSI)). **c)** Left: There was no effect of age or housing on the Amplitude of sEPSC. Right: There was a significant decrease between p35 and p60 in sIPSC amplitude with no effect of housing (sEPSC: 2-way ANOVA, age factor: $F(1,81) = 1.55$, $p = 0.22$, housing factor: $F(1,81) = 1.29$, $p = 0.26$, interaction: $F(1,81) = 0.11$, 0.74; sIPSC: 2-way ANOVA, age factor: $F(1,81) = 11.34$, $p = 0.0012$, housing factor: $F(1,81) = 0.21$, $p = 0.65$, interaction: $F(1,81) = 0.80$, $p = 0.37$, p35 sE/IPSC data: n = 20 cells from 5 mice (GH), n = 17 cells from 5 mice (jSI), adult sE/IPSC data: n=26 cells from 7 mice (GH), n=22 cells from 6 mice (jSI)). All error bars reflect +/- SEM.

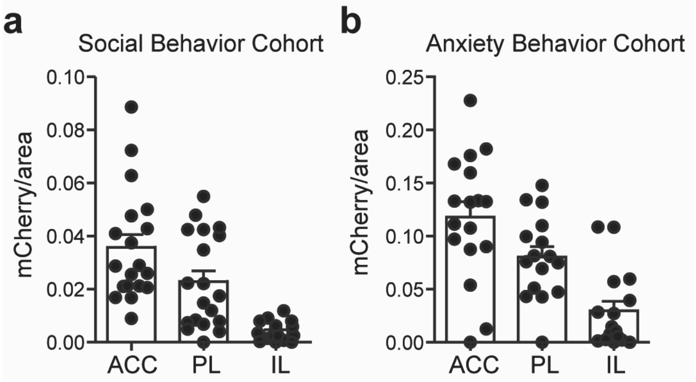

Supplementary Figure 16 (related to Fig. 6): Excitatory DREADD regional distribution. a-b) Regional distribution of mCherry indicating viral spread across the dmPFC showing expression primarily in dorsal PFC (ACC and PL) and some restricted expression in IL **a)** Social behavior cohort **b)** Anxiety behavior cohort. All error bars reflect +/- SEM.

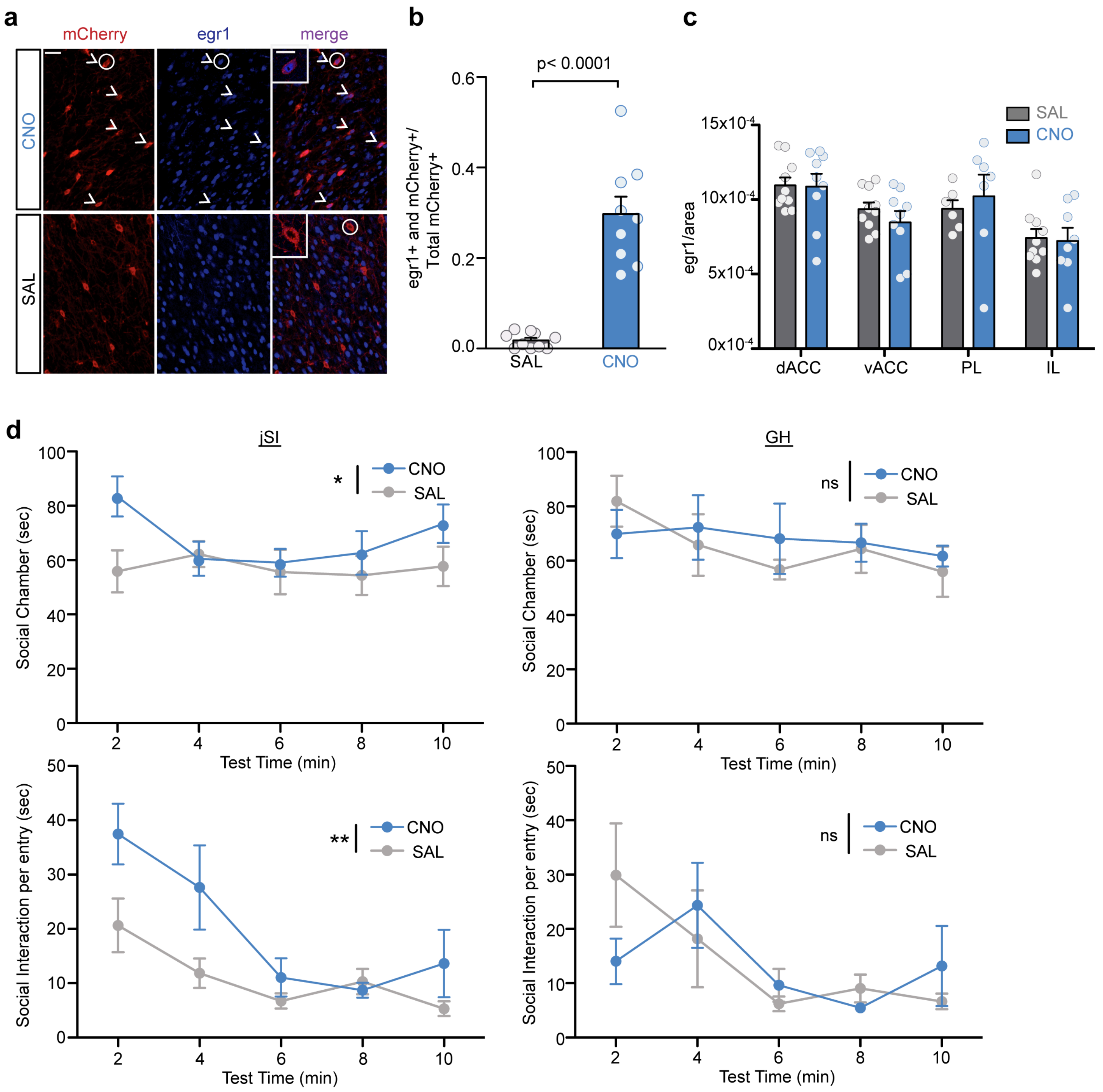

Supplementary Figure 17 (related to Fig. 6): Excitatory DREADDs reliably induce activity of dmPFC-PVIs but does not alter overall dmPFC levels of the immediate early gene egr1. a-b) Egr1 and mCherry co-staining confirmed efficacy of the eDREADD virus in activating PVIs. **a)** Representative images for CNO and SAL treated mice show significant co-localization between mCherry and egr1 only in CNO-treated mice. **b)** CNO significantly increases egr1 and mCherry co-localization in dmPFC (student's t-test, $t = 7.63$, $df = 17$, $p<0.0001$). **c)** Activating PVIs with CNO does not lead to broad changes in egr1 counts/area of dmPFC regions (2-way ANOVA, effect of drug, $F(1,61) = 0.02$, $p = 0.89$, n = 9 mice CNO, 10 mice SAL). **d)** CNO significantly

increased time spent in the social chamber and social interaction per social chamber entry across the social testing period in jSI mice (top left, time in chamber: 2 way repeated measures ANOVA, $F(1,11) = 5.7$, $p = 0.04$, $n = 12$, bottom left, social interaction per chamber entry: $F(1,11) = 11.60$, $p = 0.006$, $n = 12$) but not in GH mice (top right, time in chamber: 2 way repeated measures ANOVA, $F(1,7) = 0.08$, $p = 0.78$, $n = 8$, bottom right, social interaction per chamber entry: 2 way repeated measures ANOVA, $F(1,7) = 0.05$, $p = 0.83$, $n = 8$). All error bars reflect +/- SEM.
